# Compressive Strength Prediction of Cemented Backfill Containing Phosphate Tailings Using Extreme Gradient Boosting Optimized by Whale Optimization Algorithm

**DOI:** 10.3390/ma16010308

**Published:** 2022-12-28

**Authors:** Shuai Xiong, Zhixiang Liu, Chendi Min, Ying Shi, Shuangxia Zhang, Weijun Liu

**Affiliations:** School of Resources and Safety Engineering, Central South University, Changsha 410083, China

**Keywords:** cemented paste backfill, unconfined compressive strength, machine learning, extreme gradient boosting, WOA algorithm

## Abstract

Unconfined compressive strength (UCS) is the most significant mechanical index for cemented backfill, and it is mainly determined by traditional mechanical tests. This study optimized the extreme gradient boosting (XGBoost) model by utilizing the whale optimization algorithm (WOA) to construct a hybrid model for the UCS prediction of cemented backfill. The PT proportion, the OPC proportion, the FA proportion, the solid concentration, and the curing age were selected as input variables, and the UCS of the cemented PT backfill was selected as the output variable. The original XGBoost model, the XGBoost model optimized by particle swarm optimization (PSO-XGBoost), and the decision tree (DT) model were also constructed for comparison with the WOA-XGBoost model. The results showed that the values of the root mean square error (RMSE), coefficient of determination (R^2^), and mean absolute error (MAE) obtained from the WOA-XGBoost model, XGBoost model, PSO-XGBoost model, and DT model were equal to (0.241, 0.967, 0.184), (0.426, 0.917, 0.336), (0.316, 0.943, 0.258), and (0.464, 0.852, 0.357), respectively. The results show that the proposed WOA-XGBoost has better prediction accuracy than the other machine learning models, confirming the ability of the WOA to enhance XGBoost in cemented PT backfill strength prediction. The WOA-XGBoost model could be a fast and accurate method for the UCS prediction of cemented PT backfill.

## 1. Introduction

Cemented backfill utilizes solid waste as aggregate to prepare sustainable construction materials for use in refilling underground voids, which provides an efficient and economical approach to waste management [1,2]. The cemented backfill is prepared by homogenously mixing aggregate, hydraulic binder, and water [3,4]. After mixing, the prepared backfill slurry is transported to underground voids through pipelines, where the backfill slurry gradually solidifies and develops strength [5]. The hardened backfill provides support to the nearby rocks, reduces surface subsidence, and improves ore recovery [6,7,8]. It is reported that an unconfined compressive strength (UCS) of 0.7–2 MPa is required for the cemented backfill in typical underground mining operations [9]. Hence, sufficient UCS of cemented backfill is critical for stope safety. On the other hand, currently, determination of the UCS of cemented backfill is based on a mechanical test using a servo pressure testing machine. The mechanical test requires the preparation of backfill specimens and a rather long curing age, resulting in difficulty in obtaining the UCS values. Therefore, it is crucial for mining to create a quick and accurate method to predict the UCS of cemented backfill.

Recent advances in machine learning (ML) models could offer a method for the UCS prediction of cemented backfill. Previous studies have applied machine learning models for the strength prediction of cement-based materials, such as cement, concrete, and mortar [10,11,12]. To predict the compressive strength of self-compacted concrete, Prado-Gil et al. used a variety of machine learning models, including extreme gradient boosting (XGBoost) gradient boosting (GB), and random forest (RF). The RF model performed best [13]. To predict the compressive strength of high-performance concrete, Mosbeh et al. also created a gradient boosting regression tree model. The model demonstrated good strength prediction potential. [14]. Due to the great advantages of machine learning models, there has been a trend of using machine learning models to predict the UCS of cemented backfill in recent years [15,16]. For instance, Qi et al. combined machine learning models with multi-objective optimization in order to construct an integrated intelligent design framework to predict the UCS and slump of cemented backfill, and the model performed well in the prediction of multiple indexes [17]. In previous studies, part hyperparameters in single machine learning models were set using trial-and-error or grid search method, which could seriously reduce the accuracy of prediction. According to previous studies, optimizing the hyperparameters of a machine learning model using optimization algorithms is an effective method to improve prediction performance, which is known as a hybrid model [18,19,20]. Optimization algorithms usually include the whale optimization algorithm (WOA), the sparrow search algorithm (SSA), the particle swarm optimization algorithm (PSO), and the butterfly optimization algorithm (BOA), etc. In recent years, various hybrid models have been formed and applied in geotechnical fields, such as tunnel extrusion classification and advanced rate prediction of the tunnel [21,22,23]. Previous research has demonstrated that hybrid models outperform solely machine learningmodels. For instance, Qiu et al. applied the WOA, gray wolf optimization (GWO), and Bayesian optimization algorighms (BO) to fine-tune the hyper-parameters of the XGBoost model to predict the peak particle velocity (PPV) induced by blasting. Their results show that the performance of three optimized hybrid models was superior to the original XGBoost model [23]. Moreover, hybrid models also showed good performance in the UCS prediction of cemented backfill. Hu et al. applied the sparrow search algorithm (SSA) to optimize the extreme learning machine (ELM) to predict the UCS of cemented backfill, and the SSA-ELM model had higher prediction accuracy compared to ELM [24]. Qi et al. optimized the adaptive neuro-fuzzy inference system (ANFIS) by using the artificial bee colony (ABC) algorithm to predict the UCS of cemented backfill, and the prediction results were accurate [25]. It appears that using an optimization algorithm and a machine learning model to build a hybrid model is a feasible approach to predict the UCS of cemented backfill. However, to the authors’ knowledge, only a few studies have applied a hybrid model to UCS prediction of cemented backfill [26].

Extreme gradient boosting (XGBoost) is an innovative and efficient tree-based machine learning model proposed by Chen and Guestrin [27]. The XGBoost model can prevent the model from overfitting during training and reduce computational costs by automating parallel computation. The performance of the XGBoost model has been widely recognized in the Kaggle competition and the industrial community [28]. The WOA is an optimization algorithm that has a simple structure, few parameters, strong search ability, and simple implementation. The WOA-XGBoost model has been applied in the fields of prediction of daily reference evapotranspiration and prediction of blast-induced ground vibration [23,29]. Previous studies showed that the WOA algorithm can effectively optimize the hyperparameters of the XGBoost model and increase prediction accuracy. The WOA-XGBoost model could be a promising model for the UCS prediction of cemented backfill.

This study prepared cemented backfill using phosphate tailings (PT) as aggregate (cemented PT backfill). The WOA algorithm was used to optimize the XGBoost model in order to construct a hybrid model for the prediction of the UCS of cemented PT backfill. Five influencing factors, including the PT proportion, the OPC proportion, the FA proportion, the solid concentration, and the curing age, were used as input variables. In this paper, the UCS of backfill specimens was first determined by mechanical test. Then, the UCS results were used to build a dataset containing 63 groups. After that, RMSE, MAE, and R2 values were evaluated to determine the performance of the hybrid WOA-XGBoost model. Finally, the UCS-prediction performance of the WOA-XGBoost model was compared with the other three machine learning models (PSO-XGBoost, XGBoosst, and DT). In addition, feature importance analysis was used to obtain the importance of the input variables.

## 2. Materials and Methods

### 2.1. Materials

**Figure 1:**
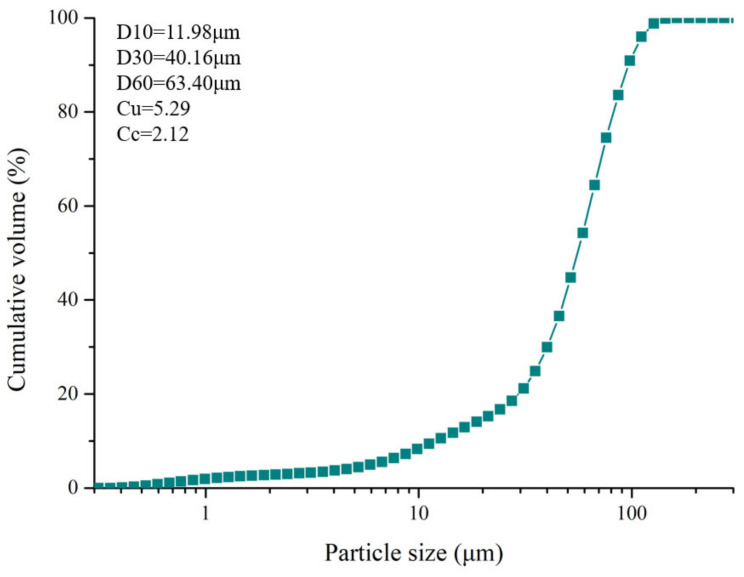
Particle size distribution of PT.

Phosphate tailings (PT) were gathered from a phosphate mine’s (Guiyang, China) tailings pond and utilized as an aggregate. The main phase compositions of PT are dolomite and quartz. Fly ash (FA, collected from a powerplant in Guiyang, China) and 42.5 ordinary Portland cement (OPC) were used as binder. Based on the Chinese standard GB/T 1596-2017, the strength activity index (A = (R/R0) × 100, where A is the strength activity index, R0 is the UCS of hardened type 42.5 ordinary Portland cement, and R is the UCS of the hardened mixture of fly ash and type 42.5 ordinary Portland cement in a 3:7 ratio) of fly ash was more than 70% after 28 days.The particle size distribution of PT (measured by a particle size analyzer, Malvern, UK) is shown in Figure 1, and the components of PT (measured by X-ray fluorescence, Bruker, Switzerland) are listed in Table 1.

### 2.2. Methods

#### 2.2.1. Preparation of Backfill Specimens

The experimental design of various ratios of raw materials and solid concentrations is shown in Table 2. The backfill slurry was prepared by mixing the PT, binder, and tap water homogeneously. The aggregate was not washed before mixing. The backfill slurry was sampled at a mixing time of 10 min, and then it was placed into models with dimensions of 40 mm × 40 mm × 40 mm. The backfill samples were taken out of the models after the final set and put into a curing box with a temperature of 20 °C and a relative humidity of 95%.

#### 2.2.2. UCS Test

The backfill specimens were removed from the curing box after curing for 7 days, 14 days, and 28 days. A servo pressure testing machine (Hualong, China) with an accuracy of ±1 N was used to determine the UCS of backfill specimens. The displacement rate was set at 0.5 mm/min. According to Chinese standard JGJ/T 70-2009, the average value of three specimens was tested for each curing age under test [30].

## 3. Results

### 3.1. Extreme Gradient Boosting Model

Extreme gradient boosting (XGBoost) is an efficient algorithm proposed by Chen and Guestrin to address supervised learning issues such as classification and regression [27]. The basic idea of the XGBoost is boosting, which uses additive training strategies and integration techniques to combine various weak learners in order to build a powerful learner [31]. To improve the computational precision, the loss function of the XGBoost model is expanded using a second-order Taylor method. Furthermore, XGboost adds a regularization term to avoid over-fitting and reduce the model complexity. The objective function of the XGBoost model consists of a term for the error function L and a term for the model complexity function Ω. The objective function can be formulated as follows:

(1) Obj=L+Ω

(2) $$L=\overset{n}{\underset{i=1}{\sum }}{\left(y_{i}-{\hat{y}}_{i}\right)}^{2}$$

(3) $$\Omega =\Upsilon T+\frac{1}{2}\lambda \overset{T}{\underset{j=1}{\sum }}\omega_{j}^{2}$$

where ${\hat{y}}_{i}$ and $y_{i}$ are the predicted and target values, ΥT is the L1 regular term,$\;\frac{1}{2}\lambda \sum_{j=1}^{T}\omega_{j}^{2}$ is the L2 regular term, *T* is the number of trees,  ω is the internal split tree weight, and Υ and λ are the regularization coefficients.

In order to minimize the objective function as much as feasible, a new function *f* was introduced to the model. The objective function can be formulated as follows:

(4) $${\mathrm{Obj}}^{\left(t\right)}=\overset{n}{\underset{i=1}{\sum }}{\left(y_{i}-\left({\hat{y}}_{i}^{\left(t-1\right)}+f_{i}\left(x_{i}\right)\right)\right)}^{2}+\Omega$$

where ${\hat{y}}_{i}^{\left(t-1\right)}$ is the predicted value of the model at time *t* − 1, and $f_{i}\left(x_{i}\right)$ is the new function added at time *t*.

A second-order Taylor expansion is used in the XGBoost technique to minimize the objective function. When the constant component is taken out, it can be seen that the objective function is only related to the error function’s first-order and second-order derivatives. The objective function can be formulated as follows:

(5) $${\mathrm{Obj}}^{\left(t\right)}=\overset{T}{\underset{j=1}{\sum }}\left[G_{j}\omega_{j}+\frac{1}{2}\left(H_{j}+\lambda \right)\omega_{j}^{2}\right]+\Upsilon T$$

where $G_{j}$ represents first-order gradient statistics on the loss function, and $H_{j}$ represents second-order gradient statistics on the loss function [32].

### 3.2. Whale Optimization Algorithm

The Whale optimization algorithm (WOA) is a metaheuristic optimization algorithm created by Mirjalili et al. [33]. By simulating humpback whale feeding behavior, the algorithm seeks to identify the optimal target parameters. It is known that humpback whales use a special behavior called the “bubble-net feeding method” when hunting. There are three phases in the whole hunting process, namely encircling prey, the bubble-net attacking method (exploitation phase), and searching for prey (exploration phase). In this study, WOA was applied in order to optimize the hyperparameters of the XGBoost model for the UCS prediction of cemented PT backfill.

#### 3.2.1. Encircling Prey

Each humpback whale symbolizes an individual, and the position of each individual in the search area indicates a solution. Humpback whales can identify the location of prey by echolocation, and all humpback whales, as a group, share information about the location of prey. The humpback whale closest to the prey position is chosen as the best candidate, after which the other humpback whales move close to that whale and tighten the envelope. A mathematical model of this behavior is described as follows:

(6) $$\vec{X}\left(t+1\right)=\vec{X^{*}}\left(t\right)-\vec{A}\cdot \left|\vec{C\cdot }\vec{X^{*}}\left(t\right)-\vec{X}\left(t\right)\right|$$

where *t* represents the current number of iterations, $\vec{X}\left(t\right)$ represents the position vector at the *t*-th iteration, and $\vec{X^{*}}\left(t\right)$ represents the position vector of the best solution obtained so far. A and C are the coefficient vectors, which can be formulated as follows:

(7) $$\vec{A}=2\vec{a\cdot }\vec{r}-\vec{a}$$

(8) $$\vec{C}=2\vec{r}$$

where $\vec{r}$ represents a random vector with values ranging from 0 to 1; $\vec{a}\;$ is a convergence factor, linearly decreasing from 2 to 0.

#### 3.2.2. Bubble-Net Attacking Method

When using a bubble net to trap prey, humpback whales assault their prey by spiraling upward and continually contracting the net. [34]. To describe this behavior graphically, two mathematical methods (shrinking encircling mechanism and spiral updating position) are designed. The shrinking encircling mechanism is achieved by decreasing the value of $\vec{a}$.

The spiral updating position phase can be formulated as follows:

(9) $$\vec{X}\left(t+1\right)={\vec{X}}_{best}\left(t\right)+\vec{D\cdot }e^{bl}\cdot \cos\left(2\pi l\right)$$

(10) $$\vec{D}=\left|{\vec{X}}_{best}\left(t\right)-\vec{X}\left(t\right)\right|$$

where $\vec{D}$ represents the distance from the i-th whale to the target prey, *b* is a constant that determines the logarithmic spiral’s shape, and *l* is a random number in the range [−1, 1].

To simulate the simultaneous behaviors of the shrinking encircling mechanism and the spiral updating position, a 50% probability of choosing between the two behaviors is assumed. The mathematical model can be expressed as follows:

(11) $$\vec{X}\left(t+1\right)=\left\{\begin{array}{c} {\vec{X}}_{best}\left(t\right)-\vec{A\cdot }\left|\vec{C\cdot }{\vec{X}}_{best}\left(t\right)-\vec{X}\left(t\right)\right|\;,p<0.5\\ {\vec{X}}_{best}\left(t\right)+\vec{D}\cdot e^{bl}\cdot \cos\left(2\pi l\right)\;,p\geq 0.5 \end{array}\right.$$

where *p* is a random number in [0, 1].

#### 3.2.3. Search for Prey

In the exploration phase, WOA updates the position of the individual to a random whale in order to implement a global search. The process can be formulated as follows:

(12) $$\vec{X}\left(t+1\right)={\vec{X}}_{rand}-\vec{A}\cdot \left|\vec{C}{\vec{\cdot X}}_{rand}-\vec{X}\right|$$

where ${\vec{X}}_{rand}\;$ is a random position vector chosen from the current population [33].

### 3.3. WOA-XGBoost Model

**Figure 2:**
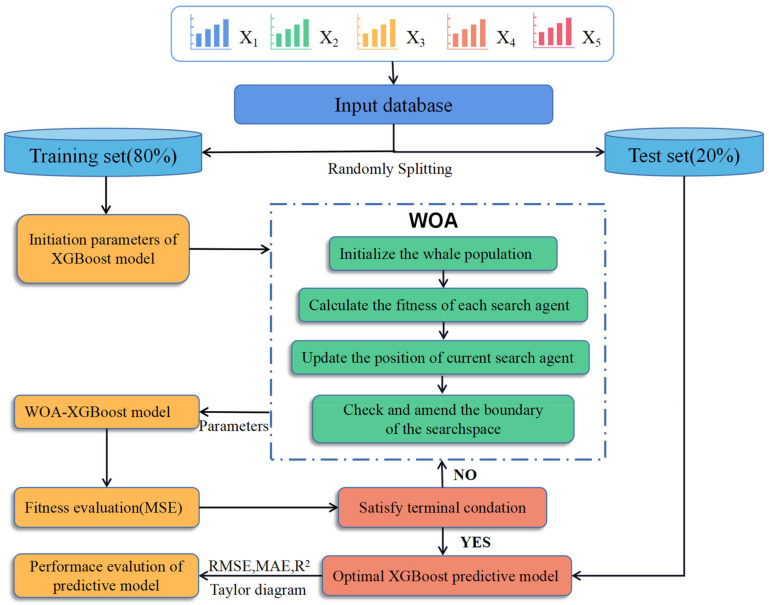
The analysis process of the WOA-XGBoost model.

In the XGBoost model, hyperparameters could have considerable influence on the prediction performance of the model [35,36,37]. The grid search method is the most-used method for the adjustment of hyperparameters. However, the search range of the grid search method is too narrow to find the optimal hyperparameters [38]. In this study, the hyperparameters of the XGBoost model were optimized by WOA; thus, the hybrid model WOA-XGBoost was proposed. Figure 2 shows the analysis process of the WOA-XGBoost model. Each whale’s position vector was determined by the XGBoost model’s parameters (learning rate, number of boosting rounds, and regular lambda). WOA outputs the final parameters of the XGBoost model after iteratively searching for the algorithm’s global optimal location. The mean squared error of attrition (MSE) is displayed at its lowest level in the optimal XGBoost model.

### 3.4. The Process of WOA-XGBoost Modeling

To develop the WOA-XGBoost model, an initial XGBoost model was developed. Then, the relevant parameters of the WOA were set. Eight different population sizes (25, 50, 75, 100, 125, 150, 175, and 200) were selected during the model development to find the optimal population size. In this study, 63 sets of data obtained from the UCS test were used as the input database. Fly ash proportion, cement proportion, phosphate tailings proportion, solid concentration, and curing age were selected as input variables. The UCS of cement PT backfill was used as the output variable. According to the Pareto principle, the original data were randomly split into a training set and a test set in the ratio of 8:2. The WOA-XGBoost model was trained using 50 sets of data from the training set, and its prediction performance was assessed using 13 sets of data from the test set. The overcomplication of ML models could result in overfitting. A 5-fold cross-validation method was used to overcome overfitting in this study. The training set was divided into 5 equal subsets, which were selected in turn for tests. The remaining four subsets were used for training to ensure that each subset had the opportunity to train and validate the model. For each training and test cycle, the cross-validation error was calculated and used to incrementally change the model parameters. Potential errors due to uneven data distribution or other unexpected circumstances were eliminated by a 5-fold cross-validation, and the robustness of each integrated learning model was ensured.

### 3.5. Evaluation Methodology

The dependability of the hybrid model is assessed using three statistical performance metrics: coefficient of determination (R^2^), mean absolute error (MAE), and root mean square error (RMSE). RMSE, MAE, and R^2^ are calculated using Equations (13)–(15), respectively, as follows:

(13) $$\mathrm{RMSE}=\sqrt{\frac{\sum_{i=1}^{N}{\left(y_{j}-y_{i}\right)}^{2}}{N}}$$

(14) $$\mathrm{MAE}=\frac{\sum_{i=1}^{N}\left|y_{j}-y_{i}\right|}{N}$$

(15) $$R^{2}=1-\frac{\sum_{i=1}^{N}{\left(y_{j}-y_{i}\right)}^{2}}{\sum_{i=1}^{N}{\left(y_{j}-\bar{y}\right)}^{2}}$$

where N is the number of samples; $y_{j}$ is the actual value; $y_{i}$ is the predicted value, and $\bar{y}$ is the average of the predicted values. The units of RMSE and MAE are Mpa, and R^2^ is expressed as a percentage. A hybrid model with lower RMSE and MAE and higher R^2^ could exhibit a better prediction performance.

## 4. Results and Discussion

### 4.1. UCS Development

**Figure 3:**
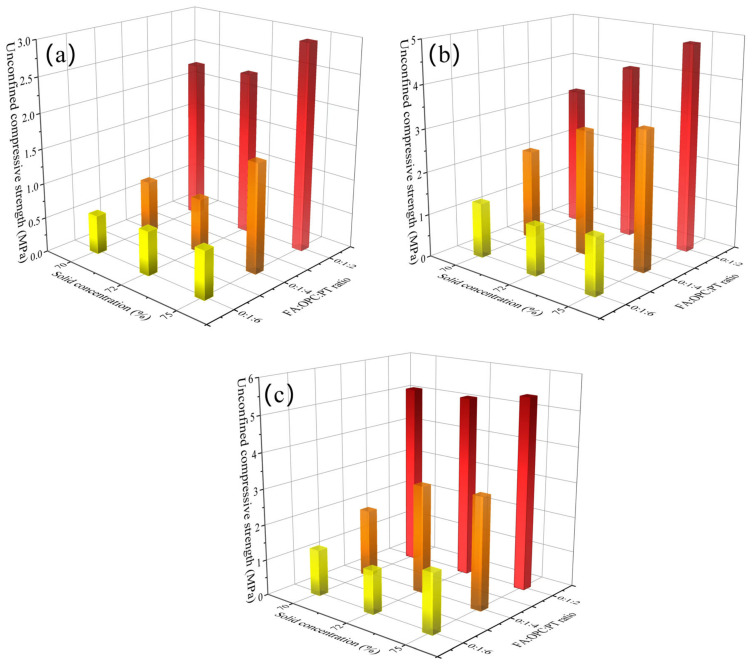
Variation of the UCS of backfill specimens with FA:OPC:PT ratio and solid concentration (using sole OPC as the binder): (**a**) 7 days; (**b**) 14 days; (**c**) 28 days.

**Figure 4:**
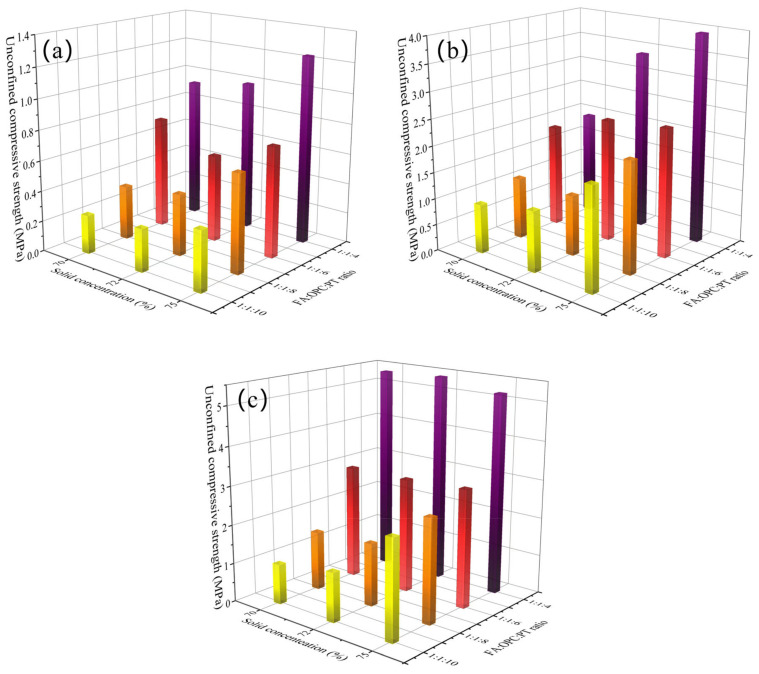
Variation of the UCS of backfill specimens with FA:OPC:PT ratio and solid concentration (using FA and OPC as the binder): (**a**) 7 days; (**b**) 14 days; (**c**) 28 days.

Figure 3 and Figure 4 show the UCS of backfill specimens. As expected, the UCS of all backfill specimens increased with curing age. For instance, the UCS of backfill specimens (FA:OPC:PT = 0:1:2, solid concentration of 70%) increased from 2.30 MPa to 5.15 MPa when the curing age was extended from 7 days to 28 days. The further hydration of the binder with curing age contributed to the development of UCS. Moreover, it appears that the backfill specimens with a higher binder/aggregate ratio gained a higher rate of UCS development over a curing age from 7 to 28 days.

It was observed that the UCS is positively related to the binder/aggregate ratio and solid concentration. As shown in Figure 3a, when the solid concentration increased from 70% to 75%, the UCS of backfill specimens with an FA:OC:PT ratio of 0:1:2, 0:1:4, and 0:1:6 increased by 28.7%, 102.6%, and 23.2%, respectively. The refinement of pores by high solid concentration could be the reason for the increase in UCS value [39]. Moreover, when the FA:OPC:PT ratio varied from 0:1:6 to 0:1:2, Figure 3a shows that the UCS of the backfill samples with solid concentrations of 70%, 72%, and 75% increased by 4.1 times, 3.67 times, and 4.3 times, respectively. The greater quantity of hydration products induced by a high binder/aggregate ratio resulted in a higher UCS value. It is worth noting that when the curing age was extended to 28 days, Figure 3c and Figure 4c show that the UCS values of backfill specimens with the highest binder/aggregate ratio (with FA:OPC:PT ratio in 0:1:2 and 1:1:4) were rather stable when subjected to different solid concentrations.

It appeared that the UCS of specimens is more sensitive to the FA:OPC:PT ratio than solid concentration. For instance, for the backfill specimens using a FA:OPC:PT ratio of 0:1:6, Figure 3c shows that when the solid concentration increased from 70% to 75%, the UCS only increased by 27.2%. However, when the FA:OPC:PT ratio varied from 0:1:6 to 0:1:2, the UCS of backfill specimens (solid concentration of 70%) obtained an increase of 294.3%. It seems that the gelling and filling of the pores of hydration products could significantly promote UCS development, which is more effective than the refinement of pores by high solid concentration.

### 4.2. Performance of WOA-XGBoost Model

**Figure 5:**
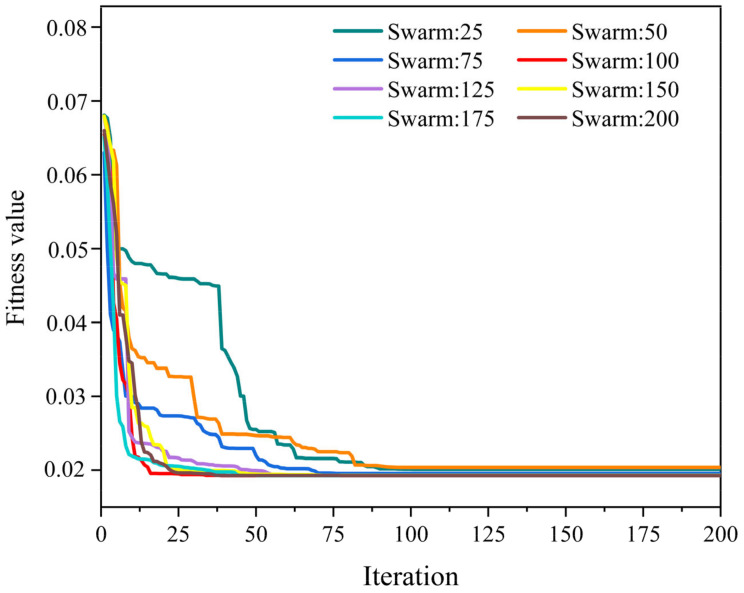
Variation of fitness value and the number of iterations of the WOA-XGBoost model with different population sizes.

**Figure 6:**
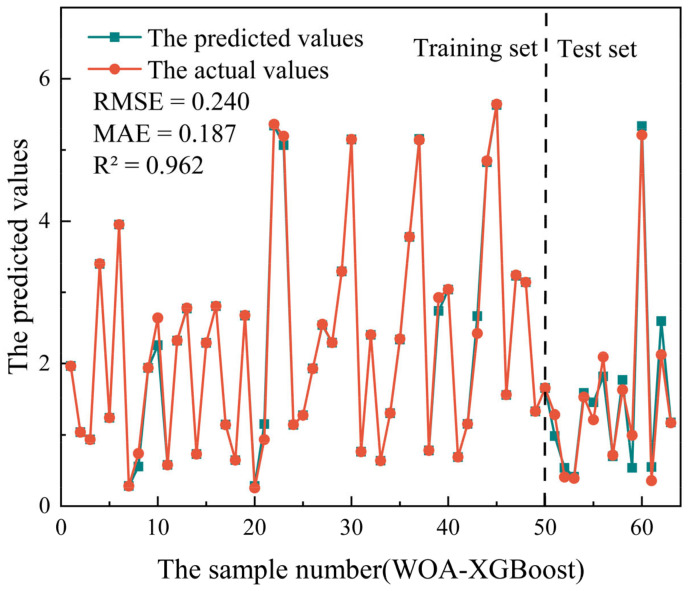
Comparison of the predicted and actual values of the WOA-XGBoost model (population size of 100).

The variation of fitness value with the number of iterations is shown in Figure 5. It was found that the fitness value of eight population values decreased dramatically as the number of iterations increased. At 100 iterations, eight population values of the WOA-XGBoost model had reached a stable level, and a stable fitness value state had been produced. After the training of the WOA-XGBoost model was complete, the calculation of the performance metrics was conducted, which were given in Table 3. It was observed that the WOA-XGBoost model with a population size of 100 gained the lowest RMSE and MAE values and the highest R^2^ value. Therefore, the population size of 100 (Num_boosting_rounds = 167, Learning_rate = 0.5235, Reg_lambda = 0.2167) was selected as the optimal parameter for the WOA-XGBoost model. Figure 6 shows a comparison of the predicted and actual values when the WOA-XGBoost model was subjected to a population size of 100. It was found that the predicted values are close to the actual values, indicating that the WOA-XGBoost model performed well in the UCS prediction of the cemented PT backfill.

### 4.3. Comparison with Machine Learning Models

The WOA-XGBoost model was compared against the PSO-XGBoost model, the original XGBoost model, and the decision tree (DT) model to further assess its performance in UCS prediction. The initial parameters of the XGBoost model and DT model were set according to the WOA-XGBoost model to ensure consistency. The initial parameters of the PSO algorithm were established by previous studies [24,26], as shown in Table 4.

**Figure 7:**
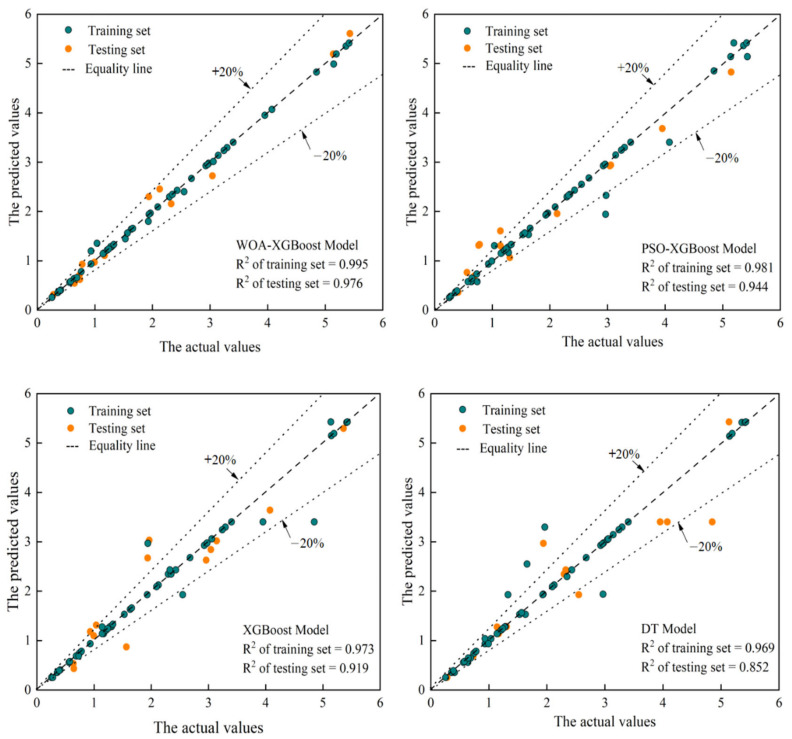
Comparison between the actual and predicted results of the four models.

**Figure 8:**
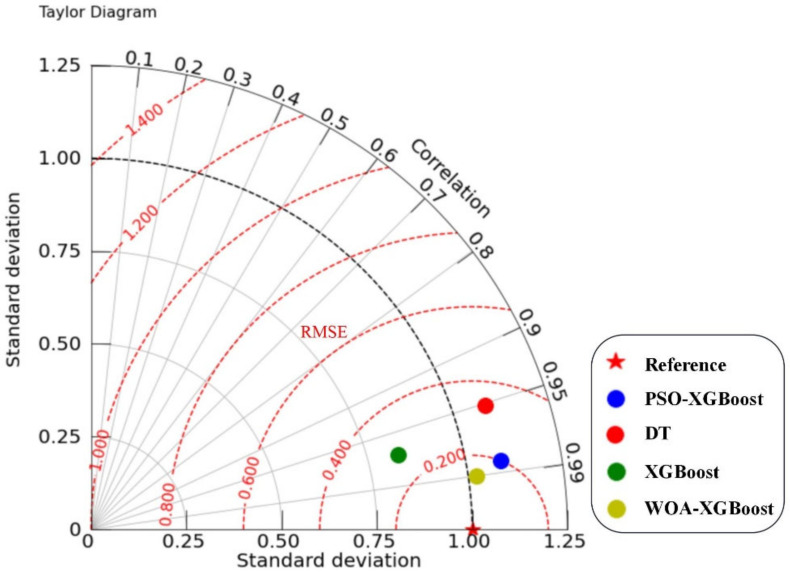
The evaluation indicators of different models.

The WOA-XGBoost model shows a better prediction performance than the remaining three models. The performance metrics for the training and test sets of the four models are shown in Table 5. Unsurprisingly, the WOA-XGBoost model had the highest R^2^ value and lowest RMSE and MAE values in both the training and test sets. Specifically, compared with PSO-XGBoost, XGBoost, and DT, the performance indexes of WOA-XGBoost on the test set showed a reduction of 37.08%, 47.86%, and 55.39% in RMSE, respectively, and 40.55%, 45.29% and 57.70% in MAE, respectively, and the R^2^ increased by 3.39%, 6.20%, and 14.55%, respectively. The actual and predicted results for the training set and test set of the four models are shown in Figure 8. It was observed that the point of the WOA-XGBoost model is located near the perfect-fitting line, indicating that the predicted values and actual values are close. On the other hand, the remaining three models exhibited larger errors (Table 5) and larger discreteness (Figure 7).

The Taylor diagram enables a more intuitive comparison of model performance than a single index of model evaluation. Taylor diagrams can present information about various models, and it has been a popular method for evaluating and testing models in recent years [32]. The RMSE, correlation coefficient, and standard deviation of the actual and predicted values were computed to further compare the prediction performance of the four models. The results are plotted as a Taylor diagram, as shown in Figure 8. Compared with other models, the WOA-XGBoost model is closer to the reference point, indicating that the WOA-XGBoost model performs better in terms of the UCS prediction of cemented PT backfill.

### 4.4. Feature Importance Analysis of Input Variables

**Figure 9:**
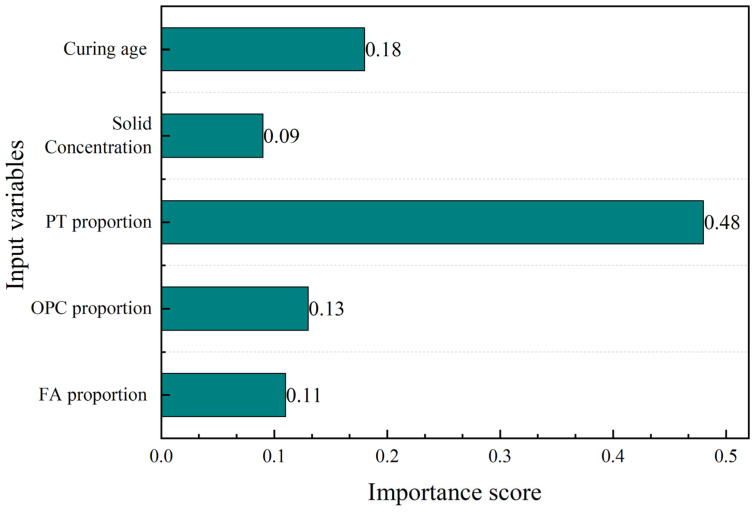
The importance scores of five input variables.

Feature importance analysis is a method used to quantify the correlation between input variables and the model [40]. The predicted results are more sensitive to the input variables with higher importance scores. Figure 9 shows the importance scores of five input variables in the WOA-XGBoost model. It can be seen that the PT proportion had the highest importance score, followed by curing age, OPC proportion, FA proportion, and solid concentration.

PT proportion, OPC proportion, and FA proportion belong to the factor of proportions of raw materials. The importance score of PT proportion, OPC proportion, and FA proportion was 0.48, 0.13, and 0.11, respectively. The total importance score of the proportions of raw materials was 0.72, which could be regarded as the most significant factor affecting the UCS of cemented PT backfill. Moreover, the importance score of the PT proportion gained the highest value among these three input variables, indicating that the binder/aggregate ratio had a greater effect on UCS than the OPC/FA ratio.

The importance score of the curing age in WOA-XGBoost obtained a value of 0.18, which meant that the effect of curing age on UCS cannot be ignored in cemented PT backfill. The experiment results in this study (Figure 3 and Figure 4) also showed that the UCS of backfill specimens could gain an increase of 83.4–552.2% over a curing age of 7 days to 28 days.

The solid concentration obtained the lowest value of importance score, which was only 0.09. The result was quite consistent with the UCS results (Figure 3 and Figure 4), which showed that the FA:OPC:PT ratio had a more significant effect on the UCS of backfill specimens than solid concentration. This phenomenon also was noticed by a previous study, which showed that the solid concentration has a smaller effect on UCS compared with the binder/aggregate ratio and curing age [17].

## 5. Conclusions

In this study, the XGBoost model was optimized using the WOA algorithm for the UCS prediction of cemented PT backfill. A total of 63 sets of experimental data were obtained through the mechanical test, and 5 variables, including fly ash percentage, cement percentage, phosphate tailings percentage, solid concentration, and curing age were used as input variables. The prediction performance was evaluated and the feature importance of input variables was calculated. The following conclusions can be drawn:

1. The WOA-XGBoost prediction model had high accuracy for the UCS prediction of cemented PT backfill. Compared with PSO-XGBoost, XGBoost, and DT, the prediction results of WOA-XGBoost showed a 37.08%, 47.86%, and 55.39% reduction in RMSE, 40.55%, 45.29%, and 57.70% reduction in MAE, 3.39%, 6.20%, and 14.55% improvement in R^2^, respectively. The results indicated that the prediction performance of the XGBoost model can be greatly improved by the WOA algorithm.
2. The results of the feature importance analysis showed that PT proportion was the most important input variable, followed by curing age, OPC proportion, FA proportion, and solid concentration. The importance score of the PT proportion was 0.48, and the total importance score of the proportions of raw materials was 0.72, indicating that the binder/aggregate ratio was the key to obtaining sufficient UCS for cemented PT backfill.
3. WOA-XGBoost model could provide a promising method for the UCS prediction of cemented PT backfill. Therefore, the model can facilitate mine production. The model achieved better performance than other machine learning models and demonstrated potential for use in other geotechnical applications. In the future, with the addition of more training data, the performance of the WOA-XGBoost model may be more accurate.

## Figures and Tables

**Table 1:** Main chemical components of FA, OPC, and PT.

Chemical Components	FA (%)	OPC (%)	PT (%)
SiO_2_	51.41	29.00	61.10
CaO	4.38	45.12	18.74
P_2_O_5_	0.15	0.28	8.80
MgO	0.54	2.85	5.61
Fe_2_O_3_	3.82	5.70	0.86
Al_2_O_3_	35.17	0.01	0.83
SO3	1.30	3.31	0.67
K_2_O	1.18	1.35	0.62
F	0.00	0.00	0.50

**Table 2:** Experimental design.

Name	FA:OPC:PT Ratio	Solid Concentration
T1	0:1:2	70%
T2	0:1:4	70%
T3	0:1:6	70%
T4	0:1:2	72%
T5	0:1:4	72%
T6	0:1:6	72%
T7	0:1:2	75%
T8	0:1:4	75%
T9	0:1:6	75%
T10	1:1:4	70%
T11	1:1:6	70%
T12	1:1:8	70%
T13	1:1:10	70%
T14	1:1:4	72%
T15	1:1:6	72%
T16	1:1:8	72%
T17	1:1:10	72%
T18	1:1:4	75%
T19	1:1:6	75%
T20	1:1:8	75%
T21	1:1:10	75%

**Table 3:** The performance indexes of the WOA-XGBoost model with different population sizes.

Swarm Size	Training Set			Test Set		
	R^2^	RMSE	MAE	R^2^	RMSE	MAE
25	0.983	0.174	0.151	0.95	0.344	0.217
50	0.989	0.169	0.136	0.964	0.272	0.244
75	0.987	0.171	0.139	0.955	0.344	0.274
100	0.995	0.156	0.114	0.976	0.207	0.151
125	0.992	0.165	0.120	0.973	0.246	0.191
150	0.991	0.179	0.135	0.966	0.279	0.222
175	0.987	0.171	0.139	0.959	0.33	0.239
200	0.984	0.173	0.145	0.955	0.349	0.258

**Table 4:** The initial parameters of the PSO algorithm.

Population Size	Maximum Number of Iterations	Local Learning Factor	Global Learning Factor	The Proportionality Constant of the Rate
50	100	1.8	1.8	0.6

**Table 5:** Performance indexes of WOA-XGBoost model, PSO-XGBoost model, XGBoost model, and DT model.

Model	Training Set			Test Set		
	R^2^	RMSE	MAE	R^2^	RMSE	MAE
WOA-XGBoost	0.995	0.156	0.114	0.976	0.207	0.151
PSO-XGBoost	0.981	0.201	0.153	0.944	0.329	0.254
XGBoost	0.973	0.246	0.191	0.919	0.397	0.276
DT	0.969	0.276	0.215	0.852	0.464	0.357

## Data Availability

Data will be made available on request.

## References

[1] Ercikdi B., Kulekci G., Yilmaz T. (2015). Utilization of Granulated Marble Wastes and Waste Bricks as Mineral Admixture in Cemented Paste Backfill of Sulphide-Rich Tailings. Constr. Build. Mater..

[2] Ma D., Kong S., Li Z., Zhang Q., Wang Z., Zhou Z. (2021). Effect of Wetting-Drying Cycle On Hydraulic and Mechanical Properties of Cemented Paste Backfill of the Recycled Solid Wastes. Chemosphere.

[3] Lu G., Fall M., Cui L. (2017). A Multiphysics-Viscoplastic Cap Model for Simulating Blast Response of Cemented Tailings Backfill. J. Rock Mech. Geotech. Eng..

[4] Min C., Shi Y., Liu Z. (2021). Properties of Cemented Phosphogypsum (Pg) Backfill in Case of Partially Substitution of Composite Portland Cement by Ground Granulated Blast Furnace Slag. Constr. Build. Mater..

[5] Qi C., Chen Q., Dong X., Zhang Q., Yaseen Z.M. (2020). Pressure Drops of Fresh Cemented Paste Backfills through Coupled Test Loop Experiments and Machine Learning Techniques. Powder Technol..

[6] Cihangir F., Ercikdi B., Kesimal A., Ocak S., Akyol Y. (2018). Effect of Sodium-Silicate Activated Slag at Different Silicate Modulus On the Strength and Microstructural Properties of Full and Coarse Sulphidic Tailings Paste Backfill. Constr. Build. Mater..

[7] Ercikdi B., Kesimal A., Cihangir F., Deveci H., Alp I. (2009). Cemented Paste Backfill of Sulphide-Rich Tailings: Importance of Binder Type and Dosage. Cem. Concr. Compos..

[8] Fall M., Benzaazoua M., Saa E.G. (2008). Mix Proportioning of Underground Cemented Tailings Backfill. Tunn. Undergr. Space Technol..

[9] Brakebusch F.W. (1994). Basics of Paste Backfill Systems. Min. Eng..

[10] Li Q., Song Z. (2022). High-Performance Concrete Strength Prediction Based On Ensemble Learning. Constr. Build. Mater..

[11] Imran H., Ibrahim M., Al-Shoukry S., Rustam F., Ashraf I. (2022). Latest Concrete Materials Dataset and Ensemble Prediction Model for Concrete Compressive Strength Containing Rca and Ggbfs Materials. Constr. Build. Mater..

[12] Sun Y., Li G., Zhang N., Chang Q., Xu J., Zhang J. (2021). Development of Ensemble Learning Models to Evaluate the Strength of Coal-Grout Materials. Int. J. Min. Sci. Technol..

[13] De-Prado-Gil J., Palencia C., Silva-Monteiro N., Martínez-García R. (2022). To Predict the Compressive Strength of Self Compacting Concrete with Recycled Aggregates Utilizing Ensemble Machine Learning Models. Case Stud. Constr. Mater..

[14] Kaloop M.R., Kumar D., Samui P., Hu J.W., Kim D. (2020). Compressive Strength Prediction of High-Performance Concrete Using Gradient Tree Boosting Machine. Constr. Build. Mater..

[15] Yu Z., Shi X., Chen X., Zhou J., Qi C., Chen Q., Rao D. (2021). Artificial Intelligence Model for Studying Unconfined Compressive Performance of Fiber-Reinforced Cemented Paste Backfill. Trans. Nonferrous Met. Soc. China.

[16] Xiao C., Wang X., Chen Q., Bin F., Wang Y., Wei W., Yilmaz E. (2020). Strength Investigation of the Silt-Based Cemented Paste Backfill Using Lab Experiments and Deep Neural Network. Adv. Mater. Sci. Eng..

[17] Qi C., Chen Q., Sonny Kim S. (2020). Integrated and Intelligent Design Framework for Cemented Paste Backfill: A Combination of Robust Machine Learning Modelling and Multi-Objective Optimization. Miner. Eng..

[18] Zhou J., Huang S., Qiu Y. (2022). Optimization of Random Forest through the Use of Mvo, Gwo and Mfo in Evaluating the Stability of Underground Entry-Type Excavations. Tunn. Undergr. Space Technol..

[19] Zhang P., Yin Z., Jin Y., Chan T.H.T., Gao F. (2021). Intelligent Modelling of Clay Compressibility Using Hybrid Meta-Heuristic and Machine Learning Algorithms. Geosci. Front..

[20] Li E., Yang F., Ren M., Zhang X., Zhou J., Khandelwal M. (2021). Prediction of Blasting Mean Fragment Size Using Support Vector Regression Combined with Five Optimization Algorithms. J. Rock Mech. Geotech. Eng..

[21] Yang H., Wang Z., Song K. (2022). A New Hybrid Grey Wolf Optimizer-Feature Weighted-Multiple Kernel-Support Vector Regression Technique to Predict Tbm Performance. Eng. Comput..

[22] Chang Q., Zhou H., Hou C. (2008). Using Particle Swarm Optimization Algorithm in an Artificial Neural Network to Forecast the Strength of Paste Filling Material. J. China Univ. Min. Technol..

[23] Qiu Y., Zhou J., Khandelwal M., Yang H., Yang P., Li C. (2022). Performance Evaluation of Hybrid Woa-Xgboost, Gwo-Xgboost and Bo-Xgboost Models to Predict Blast-Induced Ground Vibration. Eng. Comput..

[24] Hu Y., Li K., Zhang B., Han B. (2022). Strength Investigation of the Cemented Paste Backfill in Alpine Regions Using Lab Experiments and Machine Learning. Constr. Build. Mater..

[25] Qi C., Ly H., Minh Le L., Yang X., Guo L., Thai Pham B. (2021). Improved Strength Prediction of Cemented Paste Backfill Using a Novel Model Based On Adaptive Neuro Fuzzy Inference System and Artificial Bee Colony. Constr. Build. Mater..

[26] Qi C., Fourie A., Chen Q. (2018). Neural Network and Particle Swarm Optimization for Predicting the Unconfined Compressive Strength of Cemented Paste Backfill. Constr. Build. Mater..

[27] Chen T.Q., Guestrin C. Xgboost: A Scalable Tree Boosting System. Proceedings of the KDD’16: 22nd ACM SIGKDD International Conference on Knowledge Discovery and Data Mining.

[28] Chen Y., Xu Y., Jamhiri B., Wang L., Li T. (2022). Predicting Uniaxial Tensile Strength of Expansive Soil with Ensemble Learning Methods. Comput. Geotech..

[29] Yan S., Wu L., Fan J., Zhang F., Zou Y., Wu Y. (2021). A Novel Hybrid Woa-Xgb Model for Estimating Daily Reference Evapotranspiration Using Local and External Meteorological Data: Applications in Arid and Humid Regions of China. Agric. Water Manag..

[30] (2009). Standard for Test Method of Performance on Building Mortar.

[31] Sheikhi S. (2021). An Effective Fake News Detection Method Using Woa-Xgbtree Algorithm and Content-Based Features. Appl. Soft Comput..

[32] Tran V., Nguyen D. (2022). Novel Hybrid Woa-Gbm Model for Patch Loading Resistance Prediction of Longitudinally Stiffened Steel Plate Girders. Thin-Walled Struct..

[33] Mirjalili S., Lewis A. (2016). The Whale Optimization Algorithm. Adv. Eng. Softw..

[34] Zhou J., Qiu Y., Zhu S., Armaghani D.J., Li C., Nguyen H., Yagiz S. (2021). Optimization of Support Vector Machine through the Use of Metaheuristic Algorithms in Forecasting Tbm Advance Rate. Eng. Appl. Artif. Intell..

[35] Gu Z., Cao M., Wang C., Yu N., Qing H. (2022). Research On Mining Maximum Subsidence Prediction Based On Genetic Algorithm Combined with Xgboost Model. Sustainability.

[36] Su J., Wang Y., Niu X., Sha S., Yu J. (2022). Prediction of Ground Surface Settlement by Shield Tunneling Using Xgboost and Bayesian Optimization. Eng. Appl. Artif. Intell..

[37] Nguyen V., Tran V., Nguyen D., Sadiq S., Park D. (2022). Novel Hybrid Mfo-Xgboost Model for Predicting the Racking Ratio of the Rectangular Tunnels Subjected to Seismic Loading. Transp. Geotech..

[38] Song Y., Li H., Xu P., Liu D. (2022). A Method of Intrusion Detection Based On Woa-Xgboost Algorithm. Discrete Dyn. Nat. Soc..

[39] Liu L., Fang Z., Qi C., Zhang B., Guo L., Song K. (2018). Experimental Investigation On the Relationship Between Pore Characteristics and Unconfined Compressive Strength of Cemented Paste Backfill. Constr. Build. Mater..

[40] Liu K., Dai Z., Zhang R., Zheng J., Zhu J., Yang X. (2022). Prediction of the Sulfate Resistance for Recycled Aggregate Concrete Based On Ensemble Learning Algorithms. Constr. Build. Mater..

